# Advances in anterior cruciate ligament reconstruction: Risk stratification, graft choices, and functional recovery in 2026

**DOI:** 10.1002/jeo2.70766

**Published:** 2026-05-22

**Authors:** M. Enes Kayaalp, Efstathios Konstantinou, Gian A. Lucidi, Oğuzhan Ak, Natalie Mengis, Koji Nukuto, Hamit C. Kahraman, Erdem A. Sezgin, Robert Prill

**Affiliations:** ^1^ Department of Orthopaedics and Traumatology University of Health Sciences, Istanbul Fatih Sultan Mehmet Training and Research Hospital Istanbul Türkiye; ^2^ Department of Orthopaedics and Traumatology University Hospital Brandenburg/Havel, Brandenburg Medical School Theodor Fontane Brandenburg Havel Germany; ^3^ Faculty of Health Sciences Brandenburg Brandenburg Medical School Theodor Fontane Germany; ^4^ Department of Orthopaedic Surgery and Musculoskeletal Trauma University Hospital of Larisa, School of Health Sciences, University of Thessaly Larisa Greece; ^5^ II Clinica, IRCCS Istituto Ortopedico Rizzoli Bologna Italy; ^6^ Faculty of Medicine, Department of Orthopaedics and Traumatology Gazi University Ankara Türkiye; ^7^ Department of Orthopedic Surgery and Traumatology Kantonsspital Baselland Bruderholz Switzerland; ^8^ Department of Clinical Research, Research Group Michael T. Hirschmann, Regenerative Medicine & Biomechanics University of Basel Basel Switzerland; ^9^ Department of Orthopaedic Surgery Kobe University Graduate School of Medicine Kobe Japan

**Keywords:** anterior cruciate ligament reconstruction, arthroscopy, patient‐reported outcome measures, rehabilitation, risk assessment

## Abstract

**Purpose:**

To synthesize recent developments in anterior cruciate ligament reconstruction (ACLR), highlighting emerging evidence, evolving surgical strategies, and persistent controversies in risk stratification, graft selection, surgical planning and rehabilitation.

**Methods:**

A narrative review of the recent literature (2023–2026) was conducted, incorporating randomized controlled trials, systematic reviews, large registry analyses and emerging studies. The review focused on evolving concepts in graft selection, imaging‐guided surgical planning, biomechanical risk factors, neuromuscular recovery and long‐term outcomes following ACL reconstruction.

**Results:**

Quadriceps tendon autografts have gained prominence for their robust dimensions and low donor‐site morbidity, with bone‐block variants potentially enhancing rotational stability. Imaging innovations—3D mapping, navigation and individualized morphometrics—improve tunnel precision. New studies reinforce posterior tibial slope, notch shape and hyperextension as critical anatomical risk factors. Neuromuscular training techniques such as VR‐based rehabilitation, blood flow restriction, and individualized strength timelines improve functional symmetry and readiness. Registry data show rising ACLR rates, especially among young females, and link failure to younger age, high activity, delayed surgery and septic arthritis. PROMs correlate poorly with objective performance, underscoring the need for integrated assessment. Long‐term outcomes remain favourable across graft types, with no consistent differences in osteoarthritis progression.

**Conclusion:**

ACLR is transitioning toward a precision‐based, individualized model. This paradigm shift integrates anatomical risk profiling, tailored graft selection, and multimodal rehabilitation to optimize both biological and functional outcomes. Yet, unresolved questions, around pediatric predictors, repair techniques, and real‐world adherence, highlight the need for ongoing refinement. The field is evolving from reconstruction toward comprehensive restoration, grounded in anatomy, technology and behavioural science.

**Level of Evidence:**

Level V, narrative review.

**Abbreviations**
ACL: anterior cruciate ligament
ACLR: anterior cruciate ligament reconstruction
ADC: apex of the deep cartilage
ARC: Augmented Reality–Assisted Navigation (*if retained in text; otherwise remove*)
BFR: blood flow restriction
BPTB: Bone–Patellar Tendon–Bone
CT: computed tomography
DB: double‐bundle
DIS: dynamic intraligamentary stabilization
HS: Hamstring
IKDC: International Knee Documentation Committee
KNEE‐ACL: Knee Numeric‐Entity Evaluation Score—ACL Module
KOOS: Knee Injury and Osteoarthritis Outcome Score
LCL: lateral collateral ligament
LET: lateral extra‐articular tenodesis
LSI: Limb Symmetry Index
MRI: magnetic resonance imaging
OA: osteoarthritis
PCA: Posterior Femoral Cortex Angle (Posterior Cruciate Ligament–Posterior Femoral Cortex Angle)
PCL: posterior cruciate ligament
PL: peroneus longus
PLO: *(or PTS‐decreasing osteotomy, if preferred)*
PLO: Posterior Tibial Slope–Lowering Osteotomy (*if mentioned as slope‐correcting osteotomy*)
PROMs: patient‐reported outcome measures
PTOA: post‐traumatic osteoarthritis
PTS: Posterior Tibial Slope
QT: quadriceps tendon
RTS: return to sport
SB: single‐bundle
SEE: standard error of the estimate
VRBT: Virtual Reality–Based Training
WOS: Web of Science

## INTRODUCTION

Anterior cruciate ligament (ACL) injuries frequently lead to functional impairment and long‐term degeneration long‐term joint degeneration if left untreated. ACL reconstruction (ACLR) techniques have continually improved, yielding generally high rates of knee stability. Despite advances yielding good knee stability, return‐to‐sport (RTS) rates remain suboptimal (~60%), prompting ongoing innovation [80, 86]. Notably, large epidemiologic studies have observed a rising incidence of ACL tears and surgeries in youth athletes, especially young females [80]. These trends underscore the need for updated individualized approaches to improve long‐term outcomes.

Recent research has enhanced our understanding of patient‐specific risk factors for ACL injury and graft failure. Anatomical variables like a steep posterior tibial slope (PTS) or narrow notch have been shown to markedly increase ACL rupture risk [48, 118]. Likewise, younger age, high activity level, delayed surgery and modifiable factors such as smoking have emerged as significant predictors of graft failure in large cohort analyses [33, 100]. Recognizing these risk profiles allows surgeons to better stratify patients and consider adjunct procedures or tailored rehabilitation to mitigate re‐injury risk.

Modern imaging and surgical technology now enable more precise, anatomy‐guided ACLR. Three‐dimensional MRI/CT mapping and computer‐assisted navigation facilitate patient‐specific tunnel placement that closely restores native ligament anatomy [121]. This individualized approach, combined with improved intraoperative landmarks, helps address the wide variation in knee morphology and may reduce technical errors. Concurrently, graft selection has expanded beyond the traditional hamstring (HS) and patellar tendon options. Quadriceps tendon (QT) autografts in particular are gaining popularity due to their robust size and strength, studies report average quad graft diameters around 9 mm (consistently larger than HSs) with low donor‐site morbidity [19]. Also, rehabilitation protocols are evolving to address the persistent neuromuscular deficits after ACLR and safely expedite return to sport. Virtual reality‐based neuromuscular training and proprioceptive exercises have recently demonstrated improved pain relief, better knee function and enhanced dynamic balance compared to standard therapy [15]. Blood flow restriction training is another adjunct that has been validated in multiple randomized trials, it can significantly boost early quadriceps strength and functional scores while using lower loads, helping athletes regain symmetry more quickly [126].

In summary, ACL surgery is trending toward a more individualized, data‐driven paradigm [76]. Surgeons now leverage detailed imaging and patient‐specific risk profiles to tailor graft choice and surgical technique for each knee, rather than a one‐size‐fits‐all approach. Cutting‐edge rehabilitation strategies further optimize neuromuscular recovery and confidence. This narrative review synthesizes recent developments in ACLR, including risk stratification, imaging‐based planning, graft selection, and functional recovery, and discusses how these evolving concepts are translating into superior outcomes and informed decision‐making for ACL injuries in the modern era.

## METHODS

This narrative review focused on studies published between 2023 and early 2026. A structured literature search was performed using PubMed, Scopus and Web of Science with the key words ‘anterior cruciate ligament’ and ‘ACL’. Randomized controlled trials, registry‐based studies, systematic reviews and meta‐analyses were prioritized. Titles and abstracts were screened by two orthopedic surgeons experienced in ACLR, for relevance to ACL injury or reconstruction, with a focus on graft choice, imaging‐based surgical planning, anatomic and biomechanical risk factors, neuromuscular recovery and long‐term or registry‐based outcomes. Inclusion criteria were: peer‐reviewed original articles in English or synthesized evidence on the above topics. Exclusion criteria were case reports, small case series, conference abstracts, technical notes without outcome data and non–ACL‐related studies. Disagreements were resolved via discussion.

### Risk stratification and biomechanical predictors of ACL injury and graft failure

Athletic population experiences an increased rate of ACL injuries that may lead to functional impairment and long‐term cartilage degeneration. Despite advances in surgical techniques, graft failure, associated with revision surgery and increased risk of secondary meniscal injury [102], remains a major concern. Identifying risk stratification and biomechanical predictors is essential for prevention and improving outcomes.

Risk factors associated with ACL injury and graft failure include bony morphology [9, 20, 29, 38, 75, 85, 113] or knee hyperextension [22, 43]. Biomechanical contributors include abnormal muscle activation [4, 105] and altered movement patterns [50, 99]. Risk stratification is most clinically useful when risk factors are grouped into domains that inform actionable decisions (anatomy/laxity, neuromuscular loading patterns and registry‐derived patient/system predictors). To reduce redundancy and improve narrative cohesion, registry‐derived predictors (e.g., age, smoking, activity exposure and timing) are synthesized in the ‘Long‐Term Outcomes, Complications, and Registry‐Based Data’ section rather than repeated throughout the manuscript.

PTS is one of the known risk factors for ACL injury and graft failure, which is discussed in a separate section below. Other bony morphological features associated with ACL injury or graft failure include notch width, notch width index, medial tibial depth, femoral anteversion, tibial rotation and tibial spine shape [74, 84, 116]. Among male athletes, a femoral anteversion angle greater than 19 degrees measured by CT was associated with ACL injury [74]. During skeletal maturity, ACL injured knees showed smaller medial tibial spine height and a narrower notch width than matched controls [84]. In a case control study, patients who underwent ACLR bilaterally demonstrated a significantly higher internal tibial rotation morphology than those underwent unilateral ACLR. Another case‐control study using automatic bone segmentation and statistical shape modelling reported that decreased notch width, widened medial condylar width, increased convexity of the lateral tibial plateau and broad or lateralized tibial spines were associated with retears after ACLR [7]. These studies support morphology as a risk marker, but most remain observational and can be sensitive to measurement technique and sport exposure, limiting individual‐level prediction.

Knee hyperextension is another potential risk factor. In noncontact ACL injuries, athletes with secondary injury after ACLR demonstrated greater hyperextension than those without reinjury (1.9 ± 1.6° vs. 0.3 ± 3.0°, *p* = 0.007) [8]. However, contralateral knee hyperextension alone was not associated with revision surgery after ACLR with HS autografts [22], suggesting that hyperextension may act in combination with other factors. Given conflicting findings across cohorts, hyperextension should be treated as a ‘risk modifier’ (interacting with generalized laxity and graft choice) rather than an absolute contraindication in isolation.

Surgical timing can also influence outcomes. Delaying surgery more than 75 days increased graft failure risk [64], and delay longer than 40 days was associated with increased rates of intra‐articular injury [82]. Therefore, early reconstruction may help prevent secondary joint damage. However, timing associations are vulnerable to confounding by injury severity, access to care, and sport exposure while awaiting surgery; these data should support counselling/shared decision‐making rather than a universal threshold.

Biomechanical factors are increasingly recognized. A prospective cohort study found that lower HS to quadriceps strength ratio and greater knee extension strength were associated with noncontact ACL injuries in young female soccer players [105]. Another study showed that combinations of RTS status, HS strength symmetry, ACL‐RSI score and BMI were risk indicators for a second ACL injury [4]. An early peak knee abduction moment during cutting was associated with a 7.2‐fold increase in ACL injury risk [99]. In addition, professional athletes after ACLR demonstrated decreased performance in the triple cross‐over hop for distance compared with healthy professionals [50]. Clinically, these data support criteria‐based neuromuscular assessment and rehabilitation, recognizing that laboratory‐derived thresholds may require contextual interpretation before individual decisions.

**Figure 1:**
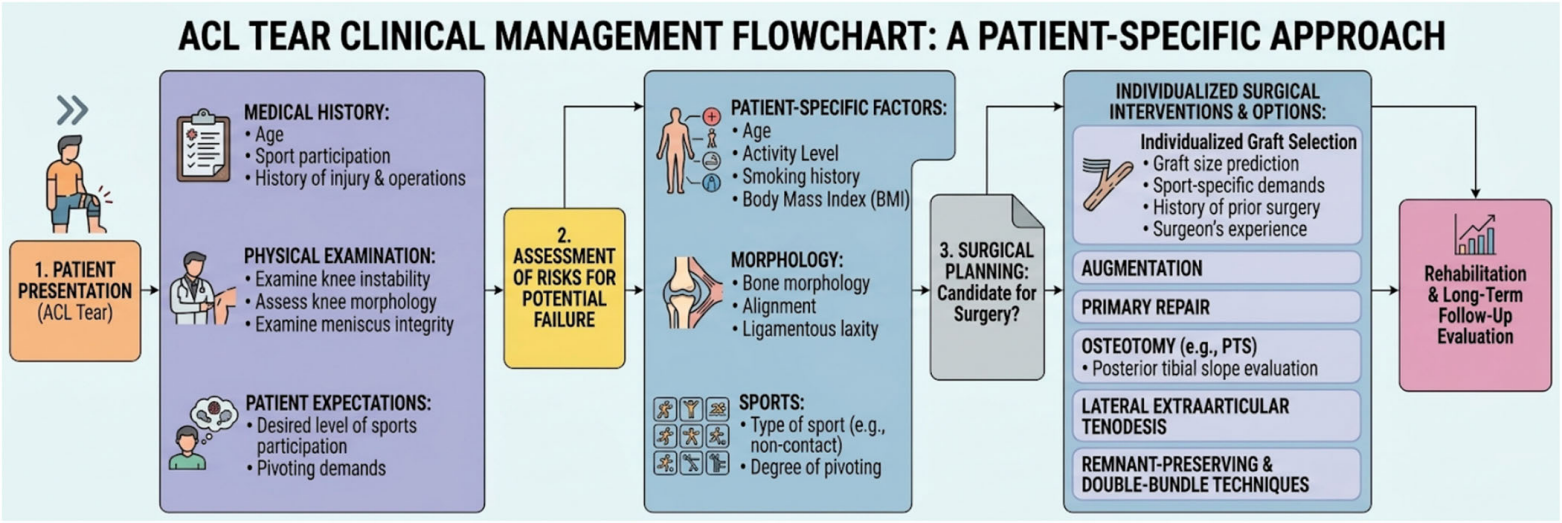
Integrated clinical algorithm for patient‐specific ACL management. This flowchart illustrates the modern paradigm of individualized anterior cruciate ligament (ACL) treatment. Following initial clinical evaluation, a comprehensive risk assessment is performed integrating patient‐specific demographic factors, knee morphology (e.g., alignment, posterior tibial slope) and specific athletic demands. These distinct risk profiles directly guide tailored surgical planning, including individualized graft selection and the consideration of adjunct procedures such as lateral extra‐articular tenodesis (LET) or slope‐reducing osteotomies for high‐risk patients. The pathway concludes with long‐term, criteria‐based rehabilitation and functional evaluation. BMI, body mass index; LET, lateral extra‐articular tenodesis; PTS, posterior tibial slope.

A comprehensive understanding of anatomical and biomechanical factors is crucial for identifying high‐risk individuals and improving graft survival. Integration of bony morphology, neuromuscular function and modifiable variables such as surgical timing may allow more personalized ACLR strategies. Integration of these domains is also the rationale for linking risk profiles to graft choice and adjunct procedures (e.g., LET) in the algorithm provided below (Figure 1).

## IMAGING, ANATOMICAL PARAMETERS AND SURGICAL PLANNING

Imaging has become indispensable in ACLR, evolving from diagnostic use to patient‐specific surgical planning, tunnel placement and postoperative assessment. Modern reconstruction strategies are shifting away from standardized approaches toward individualized anatomy‐based planning [53].

Restoring normal knee biomechanics relies heavily on accurate graft placement. MRI remains central, and newer parameters, such as the posterior cruciate ligament–posterior femoral cortex angle (PCL–PCA) and the lateral collateral ligament (LCL) sign, offer insight into chronicity and functional status. Oronowicz et al. showed lower PCL–PCA values in chronic ACL injuries, reflecting increased PCL buckling, and a positive LCL sign correlated with further decreases in PCL–PCA, suggesting progressive decompensation [77]. These findings may help identify patients in need of early reconstruction. In clinical terms, these signs may support earlier stabilization or closer monitoring when nonoperative care is considered.

Femoral tunnel precision is critical. The apex of the deep cartilage (ADC) has been validated as a reproducible arthroscopic landmark. In a 3D‐CT study, Zhang et al. demonstrated consistent tunnel positioning relative to the ADC regardless of whether the ACL remnant was preserved, with high inter‐ and intraobserver reliability [123]. Similarly, 3D‐MRI work by Lin et al. identified large variability in ACL footprint shapes, oblong in 50%, triangular in 30%, and double‐tear in 20%, supporting individualized tunnel placement [60]. Blumensaat's line morphology also affects optimal tunnel depth and height, emphasizing patient‐specific interpretation of standard radiographic landmarks [39]. The emerging clinical implication is that femoral tunnel placement should be constrained by individual footprint morphology and local landmarks rather than a single fixed coordinate.

Technology‐assisted surgery may enhance this precision. Yavari et al. reported that computer navigation and augmented‐reality tools improve spatial orientation and tunnel reproducibility, although availability and cost currently limit widespread adoption [121]. At present, the strongest rationale is improved intraoperative quality control in complex anatomy (revision cases or unusual footprints), while outcome superiority over skilled conventional technique still requires validation.

Biomechanical data highlight acceptable flexibility in tunnel angles. In porcine knees, Cheng et al. found no significant difference in anterior tibial translation, valgus angle, or graft forces between sagittal tunnel angles of 45° and 60°, allowing moderate variation when anatomic constraints are respected [11]. Finite‐element analysis by Zhang et al. further suggested that a 60° tibial drilling angle lowers proximal tibial stress, supporting its biomechanical advantage [125]. Because these data are nonclinical, they are best interpreted as permissive ‘design envelopes’ rather than rigid clinical targets.

Tunnel morphology continues to change after surgery. Lee et al. observed progressive tunnel widening over 24 months after ACLR with tibialis anterior allograft, with widening correlating to greater side‐to‐side laxity [57]. CT‐based 3D reconstructions by Liu et al. found eccentric widening, anterior/distal femoral migration and posterior/lateral tibial migration, which may complicate future revision surgery [63]. Femoral graft‐bending angle averaged 106° and did not correlate with widening or early clinical scores.

MRI graft signal characteristics further highlight the importance of tibial positioning. Park et al. reported that anteriorly placed tibial tunnels and narrow notches corresponded to higher graft signal intensity, suggesting impingement, even when femoral tunnels were anatomic [79].

Accurate measurement of bony alignment parameters can be important when considering adjunct procedures (e.g., PTS modification), but PTS is discussed in the dedicated section below to avoid repetition.

Collectively, these studies reinforce that knee morphology varies widely, influencing tunnel creation, graft behaviour and revision complexity. High‐resolution MRI and CT, 3D modelling, and navigation are driving personalized surgical planning. Key factors, including PCL angulation, footprint shape, notch dimensions, tunnel trajectory, widening risk, should be assessed in every patient. Continued validation against long‐term outcomes is needed, but the foundation for precision, anatomy‐based ACLR is firmly established.

## GRAFT SELECTION, SIZE PREDICTION AND AUGMENTATION TECHNIQUES

No single graft has proven universally superior for ACLR, and selection should be individualized based on patient morphology, sport demands, prior surgeries and surgeon experience [89]. Current clinical trends emphasize graft durability, reliable diameter, low donor‐site morbidity and RTS potential. For practical decision‐making, graft choice can be framed by two competing priorities: minimizing reinjury/revision risk in high‐risk profiles versus minimizing donor‐site morbidity and facilitating strength recovery in lower‐risk profiles.

### HS autografts

HS autografts remain common worldwide. Registry data remain conflicting: the Swedish National Knee Ligament Registry found no difference in revision risk between HS and BPTB (Bone‐Patellar Tendon‐Bone) autografts [35], whereas the New Zealand ACL Registry demonstrated higher failure rates in HS [107]. Patient factors strongly influence outcomes; individuals with generalized joint hypermobility showed a four‐fold higher rerupture risk with HS compared to BPTB [62]. In synthesis, HS remains reasonable for many patients, but caution is warranted when multiple risk modifiers coexist (e.g., high‐grade pivot shift, generalized laxity, or pronounced hyperextension), where alternative grafts or adjunct procedures may be preferable.

Graft diameter remains a major focus. A minimum 8‐mm threshold is widely recommended, and each 0.5‐mm increase reduces failure risk [40]. Newer multi‐strand configurations (six‐ or eight‐strand) reliably achieve >8 mm grafts [103]. In revision ACLR, HS autografts yield acceptable patient‐reported outcome measures (PROMs) but have similar or inferior instability and rerupture rates compared with QT or BPTB [115]. Therefore, when preoperative planning suggests a small HS graft, options include multi‐strand constructs, choosing a more size‐predictable autograft, and/or incorporating adjuncts based on overall risk rather than diameter alone.

### Bone–patellar tendon–bone autografts

BPTB autografts remain a gold‐standard option, especially for high‐demand athletes. Tomihara et al. reported higher RTS and lower rerupture rates with BPTB versus HS [109], although other meta‐analyses found no significant RTS difference [14]. Donor‐site morbidity persists; New Zealand registry data identified increased kneeling difficulty compared with HS, although overall knee pain was similar. Patellar defect bone grafting reduces residual bone void but does not significantly decrease anterior knee pain [67]. BPTB grafts are also associated with increased risk of cyclops lesions, and female sex is an independent risk factor [109]. Accordingly, BPTB offers a compelling ‘durability‐first’ profile for high‐risk athletes but requires explicit counselling regarding kneeling‐related symptoms.

### QT autografts

There is a global rise in QT studies and QT has emerged as a promising graft option due to excellent biomechanical properties and encouraging early clinical outcomes [89]. Recent comparative studies and systematic reviews report no meaningful differences in function or revision rates when comparing QT to HS or BPTB [55, 120, 122]. Meta‐analyses indicate similar RTS profiles across sexes [81].

Recent systematic reviews suggest QT commonly achieves >8 mm diameter and ≥8 cm length, implying that routine preoperative measurement may be less critical for QT than for HS planning [19].

QT harvest can be performed as soft tissue or with a patellar bone block. A large registry study of more than 1,500 patients reported similar postoperative laxity and revision rates between QT autografts harvested with or without a bone block. However, the bone‐block group demonstrated better control of rotational stability, with a residual pivot shift in 22% of patients compared with 31% in the soft‐tissue group [61]. Although the bone‐block technique carries a theoretical risk of complications, it may provide meaningful biomechanical advantages. However, in a recent study a comparison soft‐tissue QT autografts and patellar bone block QT autografts was made and revealed no statistically significant difference in revision rates and PROMs [91]. Additional advantages of the QT include reduced risk of septic arthritis and near‐complete healing of the harvest site within two years [37, 95]. Conversely, registry analyses have also reported higher short‐term revision risk with QT compared to BPTB (and similar to HS) in some settings, despite no consistent patient‐reported outcome differences; [122] this underscores shared decision‐making and registry limitations.

Evidence regarding full‐ versus partial‐thickness harvests suggests better quadriceps strength preservation with partial‐thickness QT [59]. In revision ACLR, QT has shown superior rotatory stability, improved PROMs and lower failure rates compared with HS autografts [68]. Biomechanical interest continues in graft preparation methods, such as the speed‐whip rip‐stop fixation technique, which demonstrated reduced elongation and high fixation strength in QT grafts [44].

Age‐specific studies have also been reported. In patients under 18, QT autografts showed similar clinical outcomes, revision rates and RTS compared with HS grafts [69]. QT autografts also showed promise in patients over 50, with satisfactory PROMs and good return to preinjury levels, suggesting a potential option for active individuals over 50 [70].

### Peroneus longus (PL) autografts

PL autografts have emerged as a viable alternative, consistently achieving >8 mm diameters and showing early outcomes comparable to HS with low donor morbidity [87]. Evidence remains lower‐level and shorter‐term than for established autografts; PL is best framed as an alternative option when common autografts are not available or desirable.

### Allografts

Despite convenience and lower morbidity, higher failure rates limit allograft use in young athletes. They remain reasonable options for older, lower‐demand patients and revision cases. A recent meta‐analysis found bone–soft tissue allografts have lower rerupture rates than soft‐tissue‐only allografts [104], but additional prospective studies are needed.

### Size prediction

Preoperative size estimation has clinical relevance, especially for HS grafts with risk of small diameter. MRI‐based prediction methods outperform anthropometric formulas. Minoli et al. showed that summing major and minor diameters of semitendinosus and gracilis reliably predicts four‐strand graft size without significant difference from intraoperative measurements [73]. Similarly, Vivekanatha et al. confirmed MRI‐derived cross‐sectional area as the strongest predictor of graft diameter, noting wide variability in predictive cutoffs for >8 mm [114]. Because QT is more size‐predictable in aggregate datasets, size prediction should be emphasized primarily as an HS planning tool [19].

The clinical characteristics, comparative advantages and specific patient profiles for the most common autograft options are summarized in Table 1.

**Table 1:** Comparative evidence summary of current ACL autografts.

Graft type	Key evidence & advantages	Clinical considerations & risks	Ideal patient profile
Bone–Patellar Tendon–Bone (BPTB)	Gold‐standard for stability; higher Return‐to‐Sport (RTS) and lower rerupture rates than Hamstring (HS).	Persistent donor‐site morbidity; increased kneeling difficulty; higher risk of cyclops lesions.	High‐demand/elite athletes; patients requiring maximum ‘durability‐first’ profiles.
Quadriceps Tendon (QT)	Robust diameter (avg. 9 mm); excellent biomechanics with low donor‐site morbidity.	Some registry data show higher short‐term revision risk vs. BPTB; slower quad recovery in females.	Versatile option for primary/revision cases; patients prioritizing low donor‐site pain.
Hamstring (HS)	Common global technique; multi‐strand (6–8) constructs reliably achieve >8 mm diameter.	×4 higher rerupture risk in patients with joint hypermobility; high diameter variability.	Standard primary cases; patients without generalized ligamentous laxity.
Peroneus Longus (PL)	Consistently achieves >8 mm diameter; clinical outcomes comparable to HS with low donor morbidity.	Shorter‐term and lower‐level evidence compared to traditional grafts.	Alternative autograft when established options are unavailable or undesirable.

### Augmentation techniques

To reduce rerupture rates and enable safer early rehabilitation, mechanical augmentation such as suture tape and/or internal brace augmentation and hybrid grafting has gained attention. A recent meta‐analysis found suture tape augmentation reduced graft failure and increased RTS without increasing complications [27]. Five‐year follow‐up data after HS and BPTB ACLR with internal bracing demonstrated only 1.1% failure and satisfactory PROMs [119]. Multiple studies across BPTB, QT and HS grafts confirm reduced failure and pivot shift with no adverse effects on laxity or PROMs [23, 42, 66].

Hybrid grafts (HS + allograft) are used to increase small graft diameters. However, a US cohort of patients <25 years showed no difference in revision rates between <8‐mm HS autografts and hybrid grafts >8 mm, suggesting augmentation should not be performed solely for diameter [75].

## EVOLVING SURGICAL TECHNIQUES AND INNOVATIONS

The surgical management of ACL tears has significantly evolved in recent years. Although many questions have been addressed and substantial evidence now supports individualized anatomic reconstruction, several new trends in graft selection, surgical techniques, and procedural adjuncts have gained momentum within orthopedic practice [8, 38, 89, 90, 121]. To improve interpretability, emerging techniques are summarized below with attention to evidence level, patient selection, and the definition of ‘failure’, which varies substantially across studies.

### Double‐bundle (DB) reconstruction: renewed interest

Anatomic DB ACLR was developed to better replicate native ACL fibre orientation and reduce persistent pivot shift [124]. A recent randomized controlled trial with a 5‐year follow‐up demonstrated a significantly lower failure rate in the DB group, with 7% failure compared to 23% in the single‐bundle (SB) group. Despite this advantage, DB reconstruction achieved similar clinical scores, knee stability, functional performance and osteoarthritis progression [1]. A 15‐year follow‐up study likewise found no difference between DB and SB techniques regarding osteoarthritis, with rates near 59% in both groups [97]. Thus, DB may reduce failures in selected cohorts, but the absence of consistent functional superiority supports caution when generalizing DB as a universal replacement for well‐performed anatomic SB reconstruction.

### Remnant‐preserving ACLR and ACL repair

Remnant preservation is a contemporary procedure that maintains the tibial stump rather than debriding it, with potential benefits including improved stability, enhanced proprioception and reduced failure rates [3]. A recent study reported improved graft maturation at 1 year compared with standard ACLR [98]. Precision in femoral tunnel placement remains essential. Three‐dimensional imaging studies identified the apex of the deep cartilage as a reliable landmark in remnant‐preserving techniques [123]. Remnant viability is time‐dependent. A delay of more than 12 months from injury, patient age less than 30 years, and the presence of lateral meniscus tears were associated with reduced remnant volume [106].

Modern primary ACL repair has also gained popularity. Its proposed advantages include reduced pain, preserved proprioception, avoidance of graft harvest, and simpler revision if required [12, 28]. However, the evidence remains mixed. A trial comparing dynamic intraligamentary stabilization (DIS) repair in 45 patients with healthy controls found similar functional performance, although proprioception was reduced at 1 year [31]. Another study reported a 30% failure rate at 1 year, including 7% requiring revision and 23% with persistent instability, raising concerns regarding broad clinical use [25]. However, in a larger cohort of 609 patients, the same repair technique demonstrated an 88% survival rate at 5 years, suggesting that long‐term results may depend on patient selection and surgical expertise [34]. Because reported ‘failure’ depends on technique, indication and endpoint (revision vs. laxity), the current ACL repair literature remains controversial; reconstruction should remain the reference standard for most young/high‐activity patients, while repair may be a selective option for acute tears with favourable tissue quality in older or lower‐demand profiles after explicit counselling about meaningful recurrent‐instability risk.

### Revision ACLR and associated procedures

Several advances have also been reported in revision ACL surgery [13, 108]. Lateral extra‐articular tenodesis (LET) has increased in popularity, particularly in young and high‐risk patients, as it may reduce failure rates, tunnel widening and persistent pivot shift [41]. However, outcomes in revision settings remain inconsistent. A low‐level evidence systematic review supported LET use in revision ACLR [32], but a randomized controlled trial of 103 patients with 2‐year follow‐up found no significant differences in clinical scores or laxity between revision ACLR with or without LET [101], indicating that further study is needed. This discrepancy may reflect heterogeneous indications, variable failure definitions, and limited power, emphasizing the need to interpret single studies within the broader context of evidence quality.

Optimum graft choice in revision ACLR remains debatable, albeit in limited cohorts. A recent single‐center study with 2 years follow‐up demonstated similar PROMs and revision rates with HS, BPTB and QT autografts [71].

### PTS correction: Expanding the surgical toolkit in ACL reconstruction

PTS is increasingly recognized as a biomechanical risk factor for ACL graft failure, with elevated PTS amplifying anterior tibial translation and graft stress [48]. The concept of ‘ACL zoobiquity’ has highlighted underutilization of PTS‐reducing osteotomies in human ACL reconstruction [16]. While primarily considered in revision settings, and rarely in high‐risk primary cases, PTS correction is gaining traction due to improving surgical standardization and supportive outcomes data [17, 94].

A PTS > 10.1° has been associated with significantly increased graft failure risk, informing decision‐making in revision algorithms [18] and recent evidence suggests that medial PTS ≥ 16° is predictive of multiple failures after ACL reconstruction [46]. Additionally, failed reconstructions have been linked not only to increased PTS but also to elevated posterior tibial plateau offset, reinforcing the multifactorial nature of graft failure risk [113]. PTS may also increase over time in patients undergoing multiple ACL procedures, significantly in those with medial meniscal resection, further justifying consideration in selected cases [47].

PTS correction carries risk; overcorrection can result in recurvatum or symptomatic hyperextension, making individualized planning essential. Advances such as patient‐specific cutting guides [58] and infratuberosity morphometric planning [49] could improve technical precision and may reduce complication rates.

While adult data support the association between steep PTS and ACL failure, findings in paediatric populations remain inconsistent. A recent meta‐analysis reported no clear link [26], though some studies suggest increased medial PTS in skeletally immature ACL‐injured knees [84]. PTS has also been implicated in medial meniscus posterior root tears in young athletes [52], highlighting the need for further paediatric research.

In summary, PTS‐reducing osteotomy is a valuable adjunct in revision ACL reconstruction for patients with markedly elevated PTS, provided patient selection and surgical planning are carefully individualized.

## FUNCTIONAL RECOVERY AFTER ACLR: NEUROMUSCULAR PERFORMANCE, REHABILITATION STRATEGIES AND RTS READINESS

The challenge remains in optimizing RTS after ACLR as current literature shows wide variability in RTS rates. In one study only about 60% of athletes return to their pre‐injury level, with women consistently lagging behind [21]. Elite soccer players on the other hand had a significantly higher rate, reaching above 90%, of returning to preinjury levels. A recent meta‐analysis was supportive of these high rates and reported that over 85% of athletes returned to play with nearly 90% of them reaching their preinjury level. Functional recovery depends not only on graft healing but also on neuromuscular restoration and psychological readiness. Recent research highlights persistent strength deficits, altered movement patterns and new rehabilitation strategies designed to improve RTS outcomes.

Quadriceps weakness is one of the most persistent impairments after ACLR. A systematic review reported that one in three patients still had at least 3° of knee extension loss at 1 year, improving only slightly by year 2 [93]. HS deficits are also common when tendon grafts are harvested. Patients who failed to reach approximately 77% hamstring limb symmetry index (LSI) at three months rarely achieved 90% LSI by 6 months, indicating a risk of prolonged weakness [96].

Beyond strength, altered neuromuscular control persists even in athletes cleared to return. A review of electromyography studies reported that approximately 75% of ACLR patients showed abnormal muscle activation, typically reduced quadriceps activity or compensatory HS overactivation [29]. These patterns may protect the graft but suggest incomplete recovery of normal mechanics. Movement asymmetries also become more apparent under complex or dual‐task conditions. During cognitively demanding hop tests, ACLR athletes demonstrated reduced hop distance and increased asymmetry [30]. In countermovement and drop landing tasks, force plate studies found lower jump heights and asymmetric loading, with the reconstructed limb bearing less force and the uninvolved limb compensating [56].

Explosive strength is gaining attention as a key factor in movement quality. Female athletes with higher quadriceps rate of force development landed with greater knee flexion and better shock absorption, a mechanics profile associated with lower ACL loading [36]. During double‐leg landings, a stronger explosive capacity in the ACLR limb enabled quicker achievement of peak knee extension moment and peak ground reaction force, which may aid in distributing loads efficiently [36]. However, women often demonstrate delayed quadriceps recovery, especially following QT grafts, with only 70% to 75% LSI at 12 months [20].

Rehabilitation strategies are expanding beyond traditional strengthening. Virtual reality‐based training (VRBT) has shown significant improvements in pain, strength, balance and knee scores compared with standard rehabilitation [15]. Blood flow restriction (BFR) training is another promising adjunct. A meta‐analysis including 13 randomized controlled trials reported superior quadriceps strength and Lysholm and IKDC scores in patients performing BFR training, without increases in pain or loss of motion [126]. These findings support BFR as a safe method to accelerate early muscle recovery.

Exercise prescription is also being reconsidered. Updated evidence supports the safe use of open kinetic chain knee extension when performed with appropriate load and range, improving quadriceps strength without compromising graft integrity [111]. More individualized, criteria‐based rehabilitation is replacing rigid timelines. A predictive model of 646 patients found age, sex, body mass index, sport level and preoperative quadriceps LSI were strong predictors of 1‐year strength recovery [110]. Early gains matter: reaching approximately 75%–80% quadriceps LSI within 3 months is associated with achieving 90% symmetry by 6–12 months [96].

Recent publications emphasize that structured neuromuscular training can halve ACL injury rates [85], but real‐world adoption remains limited. Coaches report time constraints and competing priorities, underscoring the need to integrate prevention exercises into routine training (e.g., as performance‐enhancing warm‐ups) and embed them in coaching curricula [85]. Programmes must also be sex‐specific: female athletes have disproportionately high ACL incidence, so prevention/rehabilitation strategies should address female anatomy, hormones, neuromuscular control and gendered training contexts to be effective [54]. Adherence is critical, only very high compliance (≈75% of sessions) yields meaningful injury reduction, therefore exercises should be made routine through coach supervision, integration into practice, and engagement tools (e.g., digital reminders) to ensure fidelity [45]. Finally, rehab protocols should tailor weight‐bearing: early full loading is safe in uncomplicated (‘green‐light’) ACLR cases, but a brief protected or limited weight‐bearing phase is advised when risk factors (e.g., meniscal repair, high tibial slope) are present to safeguard the graft without delaying functional progress [78].

In summary, modern ACL rehabilitation emphasizes individualized progression, objective testing and multimodal training. Persistent neuromuscular deficits in strength, activation, and movement quality justify the use of adjuncts such as VRBT, BFR, and carefully dosed open‐chain strengthening. A criteria‐based approach, rather than time alone, offers the best path to restoring performance and improving RTS readiness.

## LONG‐TERM OUTCOMES, COMPLICATIONS AND REGISTRY‐BASED DATA

Long‐term outcomes after ACLR are generally favourable, with sustained improvement in knee function and stability. Functional scores such as IKDC and Lysholm typically increase with longer follow‐up [10]. Post‐traumatic osteoarthritis (PTOA) remains one of the most important long‐term concerns, and its incidence is higher than in uninjured controls [6, 112]. Although some studies suggested a greater PTOA risk with BPTB grafts [65], a recent meta‐analysis found no significant difference between graft types, indicating that factors other than graft choice may drive PTOA development [112].

Outcome assessment has traditionally relied on PROMs. However, they often fail to capture objective functional capacity. A recent correlation study examining the IKDC score, Knee Numeric‐Entity Evaluation Score (KNEE‐ACL), and the Knee Injury and Osteoarthritis Outcome Score (KOOS) in relation to the Tegner Activity Scale and a battery of functional performance tests found that, with the exception of the Tegner score, PROMs show poor correlation with functional and stability tests post‐ACLR [2]. Similar findings were reported in pediatric patients, emphasizing that PROMs should not be used alone to judge recovery [117]. Patients with high PROM scores do not necessarily have lower re‐injury rates. Some may overestimate their capabilities and return to sport prematurely, while others may underestimate their readiness and delay unnecessarily [83]. A comprehensive evaluation should therefore combine PROMs with strength, balance and functional performance testing.

Although ACLR is successful for most patients, complications still occur. Risk factors for graft failure include younger age, higher activity level, delayed surgery beyond 6 months, septic arthritis, joint effusion at 3 months and tobacco use [5, 51, 92]. Active‐duty military personnel have particularly high failure rates, approaching 18% at 4 years [5]. Concomitant cartilage and meniscal lesions do not appear to compromise graft integrity [5], but registry data show that younger age, HS autograft use, medial compartment cartilage injury, low surgeon volume and transtibial drilling increase the risk of meniscal repair failure during ACLR [88]. Postoperative joint effusion may reflect synovitis from mechanical overload and is linked to reinjury [51]. Septic arthritis is especially detrimental, leading to worse PROMs and a twofold increase in graft failure risk at 5 years [92].

National registry data have expanded knowledge on ACL injury epidemiology and outcomes. Rates of ACLR have increased steadily in patients under 20 years old, with an average annual rise of 1.3% since 2002, a trend most pronounced in female athletes [80]. Similar increases have been reported in Australian Medicare registry data [9]. Large registries such as the Swedish and Norwegian Knee Ligament Registry provide valuable long‐term surveillance of surgical practice and outcomes, although incomplete reporting, selection bias and lack of rehabilitation data limit interpretation. Registry‐derived associations should be interpreted cautiously: common limitations include incomplete follow‐up, reporting bias, confounding by indication (e.g., graft choice determined by surgeon/patient factors), limited exposure data (sport participation/RTS timing), and use of revision surgery as the endpoint, which may underestimate true clinical failure. Collaboration across registries may improve completeness and generalizability of findings [24]. A recent focused review identified 13 validated strength, hop and readiness tests suitable for real‐world RTS assessment [72].

## CONCLUSION

Anterior cruciate ligament surgery continues to evolve beyond a technical procedure into a dynamic, patient‐specific strategy informed by expanding evidence. This evolution is marked by growing recognition of modifiable risk factors such as PTS, renewed interest in graft biology and augmentation, and a shift toward individualized rehabilitation protocols emphasizing neuromuscular restoration. Yet, implementation gaps, sex‐specific considerations and conflicting data—particularly in pediatric and revision settings—underscore that progress is not linear. The future of ACL surgery lies not in a singular innovation, but in the integration of biomechanical insight, biological modulation, and personalized care pathways that move the field from reconstruction to restoration.

## AUTHOR CONTRIBUTIONS

All listed authors have contributed substantially to this work: All authors have read and approved the final manuscript to be submitted and published.

## CONFLICT OF INTEREST STATEMENT

M. Enes Kayaalp: Deputy Editor‐in‐Chief of Knee Surgery, Sports Traumatology, Arthroscopy (KSSTA), Co‐editor of Joint Diseases and Related Surgery. Member of the ESSKA U‐45 Scientific Committee. Erdem Aras Sezgin: Co‐editor of Joint Diseases and Related Surgery. Robert Prill: Associate Editor of Knee Surgery, Sports Traumatology, Arthroscopy (KSSTA), paid consultant Smith and Nephew, editorial board Annals of Joint. Volker Musahl: reports educational grants, consulting fees and speaking fees from Smith & Nephew, educational grants from Arthrex and DePuy/Synthes, is a board member of the International Society of Arthroscopy, Knee Surgery and Orthopaedic Sports Medicine (ISAKOS) and deputy editor‐in‐chief of Knee Surgery, Sports Traumatology, Arthroscopy.

## ETHICS STATEMENT

The authors have nothing to report.

## Data Availability

The authors have nothing to report.
